# Utilizing Mobile Robotics for Pelvic Perturbations to Improve Balance and Cognitive Performance in Older Adults: A Randomized Controlled Trial

**DOI:** 10.21203/rs.3.rs-2997218/v1

**Published:** 2023-06-08

**Authors:** Adedeji Adeniyi, Danielle M. Stramel, Danish Rahman, Montaha Rahman, Arihant Yadav, Jingzong Zhou, Grace Y. Kim, Sunil K. Agrawal

**Affiliations:** Vagelos College of Physicians & Surgeons, Columbia University Irvine Medical Center; Columbia University; Columbia University; Columbia University; Columbia University; Columbia University; Vagelos College of Physicians & Surgeons, Columbia University Irvine Medical Center; Columbia University

## Abstract

Late-life balance disorders remain a severe problem with fatal consequences. Perturbation-based balance training (PBT), a form of rehabilitation that intentionally introduces small, unpredictable disruptions to an individual’s gait cycle, can improve balance. The Tethered Pelvic Assist Device (TPAD) is a cable-driven robotic trainer that applies perturbations to the user’s pelvis during treadmill walking. Earlier work showcased improved gait stability and the first evidence of increased cognition acutely. The mobile Tethered Pelvic Assist Device (mTPAD), a portable version of the TPAD, applies perturbations to a pelvic belt via a posterior walker during overground gait, as opposed to treadmill walking. Forty healthy older adults were randomly assigned to a control group (CG, n = 20) without mTPAD PBT or an experimental group (EG, n = 20) with mTPAD PBT for a two-day study. Day 1 consisted of baseline anthropometrics, vitals, and functional and cognitive measurements. Day 2 consisted of training with the mTPAD and post-interventional cognitive and functional measurements. Results revealed that the EG significantly outperformed the CG in cognitive and functional tasks while showcasing increased confidence in mobility. Gait analysis demonstrated that the mTPAD PBT significantly improved mediolateral stability during lateral perturbations. To our knowledge, our study is the first randomized, large group (n = 40) clinical study exploring new mobile perturbation-based robotic gait training technology.

## Introduction

Late-life balance disorders remain a severe problem with fatal consequences [1], [2]. Balance disorders can lead to impaired walking ability and the inability to adapt to the environment [3]. Abnormal walking ability and the inability to efficiently respond to instability increase the risk of falls. In the United States, falls account for the leading cause of fatal injuries among the older adults [4] and consume billions of dollars in medical costs annually [5]. Not only that, but also falls cause anxiety, reduced mobility, and frequent clinic visits for patients [6]. Consequently, resolving balance disorders while preventing falls has become a foremost priority for public health [7] among an increasingly aging US population [8].

Perturbation-based balance training (PBT) can improve balance and reduce falls among older adults [6], [9], [10], [11]. PBT is a form of rehabilitation that intentionally introduces minor, unpredictable disruptions to an individual’s gait cycle, allowing them to react and build motor skills safely. Through PBT, individuals can become more physically adept at recovering from real-life disruptive situations where they must control abrupt shifts in body weight or unexpected trips on external barriers [12], [6].

The methods of PBT implementation vary and include standing on a surface, like a thick foam, balance board, or slip board, or experiencing external forces applied by a physical therapist (PT), occupational therapist (OT), or a robotic platform [13], [14], [15]. One benefit of robotic platform PBT is the application of precise, measurable forces for specific time intervals. Utilizing robotic platforms in training enables PTs and OTs to progress and track training dosage and performance metrics systematically [16].

The robotic platforms that can provide PBT vary widely and can be categorized as proposed by Shirota et al [17]. Some devices apply perturbations at the foot level through an automated platform that changes position or orientation [18], [19], [20]) or even underfoot stiffness [21]. Soft exosuits can also apply perturbations to specific joints, like the hip joint with the SR-Hex [22]. Other platforms provide force pulses to the pelvis to study and train complete lower body reactions to these external forces. Rigid structures can apply these external forces, like that of the BAR-TM, which applies lateral perturbations [23], or the exoskeletal LOPES III, which uses anterior perturbations [24]. While these allow large forces to be applied, they add inertia to the individual, which may alter human dynamics. A robot adding more than 6 kg of mass to the user’s pelvis can significantly alter their gait [25]. Cable-driven robotic gait trainers (CDRGTs) modulate the length or tension in cables to apply forces to the pelvis, like the TPAD [26] or the CaLT [27]. These systems are typically lightweight and require multiple cables to apply forces in different directions.

**Figure 1:**
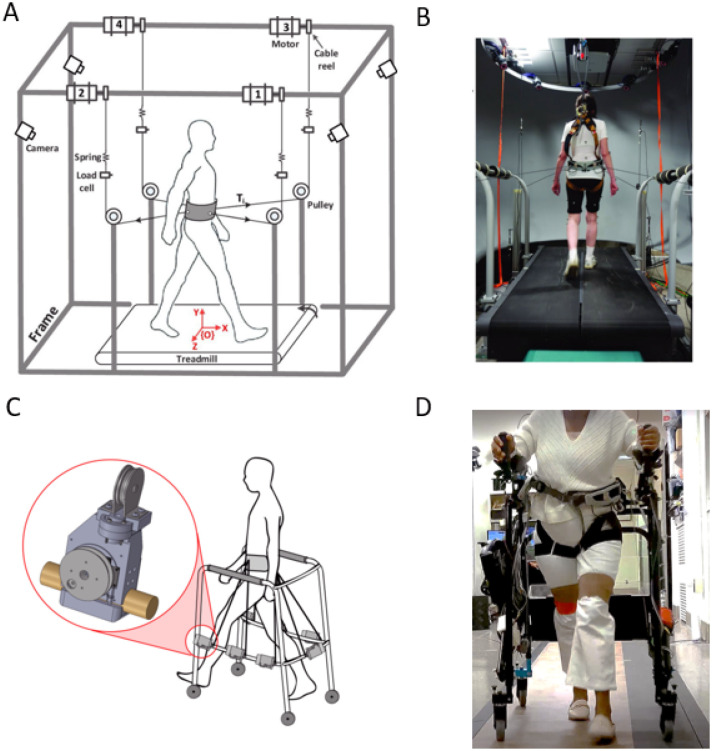
Experimental Setup. Schematics and pictures of participants walking on the TPAD are presented in panels A–B, respectively, while panels C–D show those of the mTPAD. Panel A–B and panel C are adapted with permission from Martelli et al. 2021 [10] and Stramel et al. 2020 [35], respectively.

The Robotics and Rehabilitation (RoaR) lab has developed the Tethered Pelvic Assist Device (TPAD, Fig. 1), a cable-driven robotic platform designed to apply customized forces to the pelvis for treadmill gait training, making it well suited for PBT [28]. A pilot study by Martelli et al. [10] involved the TPAD and revealed promising results. The study investigated the effectiveness of a single session of PBT and established evidence of improved gait stability with the TPAD. Participants in the experiment group, i.e., those repeatedly subjected to multidirectional perturbations during training, were better adapted to counteract timed diagonal waist-pull perturbations. The study was also the first evidence of acute benefits to cognitive performance after PBT. They proposed that the likely mechanism behind this close relationship is the cerebral cortex, the outermost area of the brain, responsible for higher cognitive functions (e.g., complex decision-making or goal-setting), short-term memory, and social behavior [29], [30], [31]. The cerebral cortex also controls compensatory responses by priming the central nervous system to perturbations [32]. As such, cognitive performance relates to sensing perturbations and evoking a rapid balance reaction in the individual [6].

The TPAD’s lightweight pelvic belt adds minimal inertia and lower limb restrictions to the user, minimally altering participants’ posture as they walk on a treadmill. However, treadmill gait differs from overground gait (walking over a natural surface, such as a sidewalk, floor, or trail) when differences in kinematic, kinetic, and electromyographic parameters are not considered [33], [34]. Given the differences between treadmill and overground gait, treadmill constraint removal is essential. To improve accessibility and allow gait training during overground walking, our lab has also developed the mobile Tethered Pelvic Assist Device (mTPAD, Fig. 1), an overground extension of the TPAD [35]. The mTPAD, which uses a posterior rollator as the frame, does not require a treadmill and is a fully contained, portable device that may be better adaptable to various locations and with the potential for offsite training, expanding accessibility. Furthermore, the mTPAD is a low-cost system that could operate in low-resource settings.

Based on the promising results of the TPAD’s PBT and the ability of the mTPAD to provide similar perturbations during overground gait, the mTPAD allows the study of how overground PBT can impact older individuals’ balance and cognition. Our pilot study investigates overground PBT’s feasibility, safety, and effectiveness with the mTPAD in healthy adults 50 and older, the age when fall risk increases [36]. Our novel study is the first randomized, large group (n = 40) clinical study investigating the potential of overground PBT using mTPAD, a CDRGT. This technical advancement would translate to a new paradigm in functional rehabilitation that could assist patients in gaining or regaining their balance.

## Methods

A functional and cognitive measures dataset was collected from a group of neurotypical older adults to evaluate the mTPAD’s potential for overground PBT. We hypothesize that a single session of PBT with the mTPAD can effectively produce acute improvements in cognitive performance and balance. Acute changes in balance were measured using (1) various functional task assessments, (2) a validated questionnaire, and (3) performance markers collected from an instrument gait mat. Additionally, vitals and anthropometric measurements further characterized functional performance. Three different screening tools and tests evaluated individuals’ cognitive function at baseline to track acute, subtle changes in cognitive function. By investigating these measures during multiple overground walking conditions, we can validate the mTPAD as a PBT tool and determine the mTPAD’s PBT effects on balance and cognition.

### Participants

Forty healthy older adults, age 50 and older and who live independently, were randomly assigned to either the control group (CG, n = 20) or the experimental group (EG, n = 20). Assignment to the EG and CG was random using computer-generated numbers. A breakdown of each group’s characteristics is detailed in the results section. The exclusion criteria included: (1) the presence of acute, severe, or unstable medical illness; (2) reporting any significant neural, muscular, or skeletal disease; and (3) inability to safely walk overground without mobility aids, e.g., cane, walker, crutches. The Columbia University Medical Center Institutional Review Board approved this study (IRB Number: AAAT7862; Expiration Date: 07/04/2023). Before beginning, study personnel informed participants of the study procedure, risks, and benefits. Participants had the opportunity to ask questions, and once the research staff answered questions, all participants signed an informed consent form. Procedures were carried out adhering to all applicable guidelines and regulations throughout.

### Procedure

**Figure 2:**
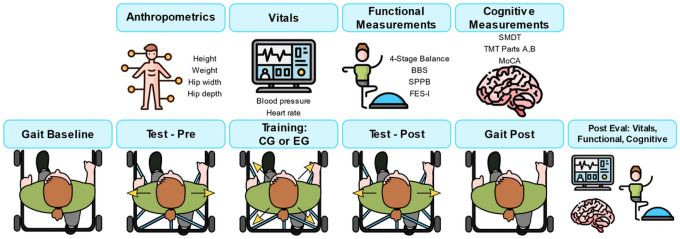
Graphical Abstract of the Experimental Protocol for Day 1 and Day 2 (top and bottom row, respectively).

Table 1 and Fig. 2 summarize the procedure for the two days participants were asked to come in. Day 1 consisted of an intake interview and collection of baseline anthropometrics, functional measurements, and cognitive measurements. Functional measures included the 4-Stage Balance test, Falls Efficacy Scale – International (FES-I), Berg Balance Scale (BBS), and Short Physical Performance Battery (SPPB). Cognitive measurements included the Montreal Cognitive Assessment (MOCA) and Trail Making Test Parts A and B (TMT-A, TMT-B). In addition, participants were assessed by the Symbol Digit Modalities Test (SMDT) with the following parameters measured: SMDT-60 seconds (SMDT-60), SMDT-90 seconds (SMDT-90), and SMDT time to complete (SMDT-C). Baseline vitals were also obtained, including systolic and diastolic blood pressure and heart rate.

Day 2 consisted of training with the mTPAD, a mobile robotic platform that applies timed forces to the pelvis [35] [37], and post-intervention cognitive and functional measurements. The research team secured mTPAD’s pelvic belt snugly at the level of the iliac crests, and the mTPAD height was self-selected between 36–41 inches.

The mTPAD experimental protocol consisted of 5 trials: Gait Baseline, Test Pre, Training, Test Post, and Gait Post. Participants walked on the Zeno Walkway at their preferred pace throughout all 5 trials. All participants had the opportunity to practice walking before the start of the trials. All trials involved 5 minutes of walking except for Training, which consisted of 10 minutes of walking. The mTPAD applied perturbations at a force equal to ~ 10% of the participant’s body weight (BW). Participants in both the EG and CG were exposed to the same conditions for Gait Baseline, Test Pre, Test Post, and Gait Post. The Training served as the intervention and differentiated the EG and CG. During Gait Baseline, participants walked without cables and without perturbations to establish the participant’s baseline walking. The research team attached cables before the start of Test Pre. Participants experienced lateral perturbations randomized by direction (left or right) and timing during the trial while walking. Next, Training was conducted with cables still attached. For Training, the mTPAD applied either no perturbations (CG) or randomized perturbations (EG). The EG experienced diagonal perturbations comprised of a combination of a lateral force (left or right) and a vertical force (up or down). Perturbations were randomized by direction and timing throughout the trial. Test Post followed the training session. As with Test Pre, the EG and CG participants experienced randomized lateral perturbations (left or right). Finally, the research team removed the cables from the pelvic brace, and participants walked unperturbed for Gait Post. Participants could take a 5-minute break after each condition. No falls, injuries, or adverse events occurred throughout the experimentation. Day 2 continued with a collection of vitals (blood pressure and heart rate). Participants completed a series of cognitive and functional tests similar to those performed on Day 1.

This study changed the perturbation forces based on the protocol condition, including lateral, superior/inferior, and diagonal in the frontal plane. These directions were selected to challenge lateral stability during overground gait. A square wave pulse was used for the perturbations for these three force conditions. The square wave had a low value of 0 Newtons, with a high value being the magnitude of the goal force applied during the experiment. The pulse width was 100 ms, and the timing between pulses was random to mitigate learning effects.

### Measures

#### Cognitive Measurements

We employed several widely used screening tools and tests to assess individuals’ baseline cognitive levels and to measure subtle, acute changes in cognitive functioning. To screen for cognitive impairment and evaluate the baseline cognitive function of individuals, we used the MOCA [38]. To detect subtle, acute changes in cognitive function before and after the mTPAD, we used the TMT and SDMT. The TMT assesses cognitive function by measuring a person’s combined visual attention, mental flexibility, and psychomotor speed [39]. The SDMT Symbol Digit Modalities measure processing and motor speed [40].

#### Functional Measurements

Our study consisted of various functional assessments, including the 4-Stage Balance test, FES-I, BBS, and SPPB. The 4-Stage Balance test assessed static postural balance [41]. The Falls Efficacy Scale-International (FES-I) is a self-report measure used to assess an individual’s confidence in their ability to perform activities without falling [42]. The BBS and SPPB are tools used to measure functional fitness and mobility in older adults. For each, scores are calculated based on the individual’s performance on each test, with higher scores indicating better functional fitness and mobility [43], [44].

#### Quantitative Measurements

As part of the non-clinical data analysis, several key performance measurement variables were collected from the Zeno Walkway and are defined in this section. These temporal, spatial, and pressure-related variables were used for the gait analysis and calculated using the ProtoKinetic software PKMAS [45]. Stride length is the distance between two successive ipsilateral heel strikes in centimeters [46]. Stride velocity is the average velocity for each stride. The single support time is the time in seconds when only one foot is in contact with the ground. The integrated pressure measurement quantitatively characterizes the contact between the ground and the foot during locomotion [47] and is calculated as the area under each footfall’s pressure curve. In addition to these parameters, the center of pressure (COP) cyclogram is investigated [48]. The COP cyclogram is a butterfly-shaped pattern used in gait analysis that illustrates COP trajectories over time during different phases of the gait cycle. The COP Cyclogram Intersection Point (CISP) is studied for both the EG and CG. The CISP change is monitored in the anterior-posterior (AP) and medial-lateral (ML) directions and measures the COP trajectory symmetry.

#### Anthropometric, Socio-Demographic, and Vitals

Anthropometric measurements included participants’ height, body weight, and hip width and depth. We also assessed the following socio-demographic factors: fall risk, education level, subjective health (5 = excellent; 4 = very good; 3 = good; 2 = fair; 1 = poor), employment status, and housing status. The vitals we obtained included systolic blood pressure, diastolic blood pressure, and heart rate. To assess fall risk, individuals who responded “yes” or affirmatively to any of the following questions were considered “at-risk” [13]:

1. Have you fallen in the past five years?
2. Have you lost your balance or almost fallen in recent memory?
3. Have you reduced your activities or changed your lifestyle because you were concerned that you might fall?
4. Do you ever feel unsteady when: getting in or out of a chair, changing direction when you are standing or walking, reaching for something above your head, or walking and talking to someone at the same time?
5. Has anyone, such as a friend or family member, expressed concern that you may have problems with your balance or are at risk of falling?

#### Statistical Analysis

All statistical analyses were performed with Stata 15 (StataCorpLP, College Station, TX, USA), Python version 3.10.8 (Python Software Foundation, Delaware, United States), or Microsoft Excel Office 365 (Microsoft Corporation, Washington, United States). Two-sided *p*-values of less than 0.05 were considered statistically significant for all tests and were defined as * : p < 0.05, ** : p < 0.01, *** : p < 0.001.

Table 2 is a descriptive analysis comparing all of the collected Day 1 baseline data (e.g., cognitive, functional, anthropometrics) between the EG and CG. Table 2 reports all continuous variables (e.g., weight, height, age) as mean ± standard deviation (SD). In contrast, the table presents categorical variables (e.g., sex, education, employment status) with the number and percentage of the total. To obtain p-values and assess differences in Day 1 baseline data between the EG and CG, a Pearson’s chi-squared test was performed for categorical variables, while a paired t-test was performed for continuous variables.

**Figure 3:**
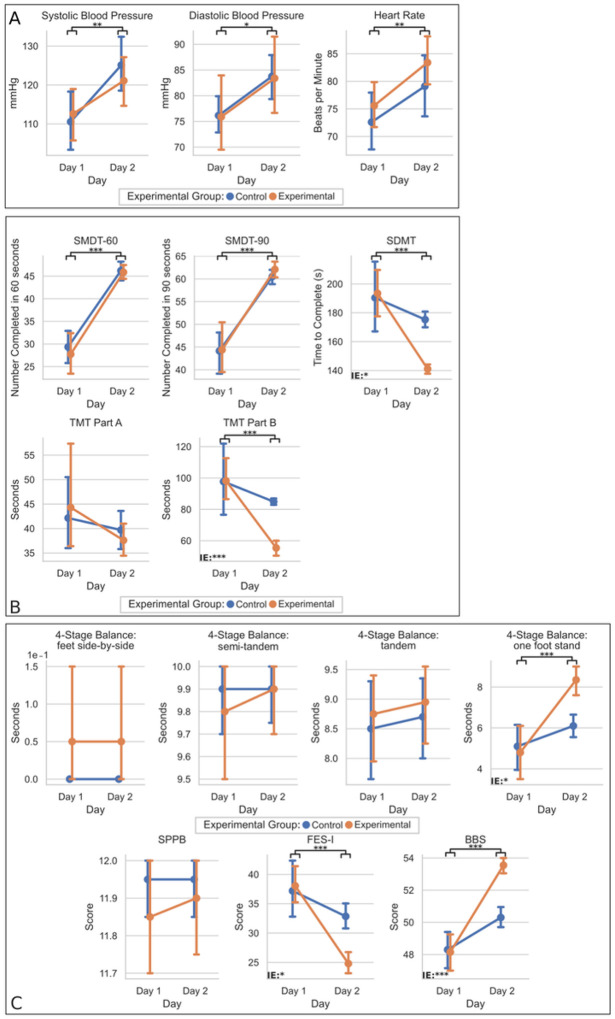
Plot of Day 1 vs. Day 2 for Vitals (Panel A), Cognitive Measurements (Panel B), and Functional Measurements (Panel C) by group (EG and CG). Statistically significant values were defined as * : p <0.05, ** : p < 0.01, *** : p < 0.001. Interaction effects indicated statistically significant main effects of the session (Day 1, Day 2)×group (EG, CG) interaction term, respectively.

Figure 3 shows the results of a two-factor mixed analysis of variance (mANOVA) comparing vitals, functional measurements, and cognitive measurements obtained on Day 1 and Day 2 for both the EG and CG. Group (EG, CG) and session (Day 1, Day 2) served as between and within-subject factors, respectively.

For the gait data, two types of analyses were used. For the comparisons between the Gait Baseline and Gait Post for the EG and CG, an mANOVA was used. An mANOVA was also used for the Test Pre and Test Post comparisons between the EG and CG. All data were checked for normality, sphericity, and homoscedasticity before applying the mANOVA. If the sphericity and homoscedasticity requirements were met, but the data were not normal, then a natural log transform was used. When comparing the training data for the EG and CG, an independent t-test was used if the data satisfied the normality and homoscedasticity conditions. If significantly not normal, a Mann-Whitney U test was used to compare the means. These tests were run for each variable’s mean and standard deviation. By investigating the changes in the standard deviation per person, we can explore the differences in variability induced by perturbation training.

## Results

### A. Demographics and Baseline

The demographics were analyzed to determine differences between the EG and CG, and shown in Table 2. The average age of participants for both groups was 71 (± 8) years old (p = 0.906). No significant difference was observed between the EG and CG in the collected Day 1 baseline data. Of note, there was no significant difference in Day 1 cognitive performance between the EG and CG, including the MOCA (p = 0.224), TMT A (p = 0.762), TMT B (p = 0.971), SMDT-C (p = 0.842), SMDT-90 (p = 0.946), and SMDT-60 (p = 0.589). In addition, no differences were observed in any Day 1 functional measurement between the EG and CG, including the FES-I (p = 0.780), BBS (p = 0.854), SPPB (p = 0.304), and all 4-Stage Balance tests: (feet side-by-side (p = 1.000); semi-tandem stand (p = 0.560); tandem stand (p = 0.681); one-foot stand (p = 0.740)).

### B. Clinical Data

Results comparing the clinical data (vitals, cognitive measurements, and functional measurements) of both days by the group are depicted in Fig. 3.

When comparing the vitals of Day 2 to those of Day 1, we observed that all individuals showed significantly elevated systolic blood pressure (F(1,1) = 9.93, p = 0.002), diastolic blood pressure (F(1,1) = 5.81, p = 0.018), and heart rate (F(1,1) = 7.64, p = 0.007) on Day 2. However, no significant interaction effects for the group-x-session were found for any of the vitals: systolic blood pressure (p = 0.411), diastolic blood pressure (p = 0.987), and heart rate (p = 0.802). This indicates that group placement did not significantly alter the vitals.

When comparing Day 2 cognitive measurements to those of Day 1, all test participants showed significantly shorter times for the TMT-B (F(1,1) = 15.15, p < 0.001), SDMT-C (F(1,1) = 18.19, p < 0.001), SDMT-90 (F(1,1) = 78.76, p < 0.001), and SDMT-60 (F(1,1) = 115.46, p < 0.001) on Day 2. No significant interaction effects for the group-x-session were found for the SDMT-90 (p = 0.726) and SDMT-60 (p = 0.148), indicating that group placement did not alter the test improvements. However, a significant interaction of group-x-session was seen for the TMT-B (p < 0.001) and SDMT-C (p = 0.021), highlighting that group placement did alter the rate of improvement between Day 1 to Day 2 and that the EG outperformed the CG in those cognitive tests. TMT-A had no significant main or interaction effect of the group-x-session, indicating that neither group placement nor session significantly impacted performance.

Regarding functional measurements, and when comparing Day 2 to those of Day 1, all individual participants showed significantly lower scores for the FES-I (F(1,1) = 27.81, p < 0.001) on Day 2. At the same time, both groups had significantly higher BBS (F(1,1) = 64.64, p < 0.001) and 4-Stage Balance: one-foot stand (F(1,1) = 20.21, p < 0.001) on Day 2. Further analysis revealed a significant interaction of group-x-session for the FES-I (p = 0.010), BBS (p < 0.001), and 4-Stage Balance: one-foot stand (p = 0.014), highlighting that group placement did alter the rate of improvement between Day 1 to Day 2 and that the EG outperformed the CG on those functional measurements. There were no significant main or interaction effects of group-x-session for SPPB (p = 0.698), 4-Stage Balance: feet side-by-side (p = 1.000), 4-Stage Balance: semi-tandem (p = 0.634), 4-Stage Balance: tandem (p = 0.609), and thus indicates that neither group placement nor session significantly affected performance.

### C. Gait Analysis

The mANOVA was repeated for two different trial combinations. The primary objective of this analysis was to determine the variance of gait-related functional parameters during and after the training. This mANOVA was conducted initially between the Gait Baseline and Gait Post trials and secondly between the Test-Pre and Test-Post trials. These trial combinations will be called the Gait and Test trials, respectively. Additionally, an unpaired t-test was conducted for the Training trials to identify any statistically significant differences between the EG and CG.

#### 1. Gait Trials (Gait Baseline vs. Gait Post)

**Figure 4:**
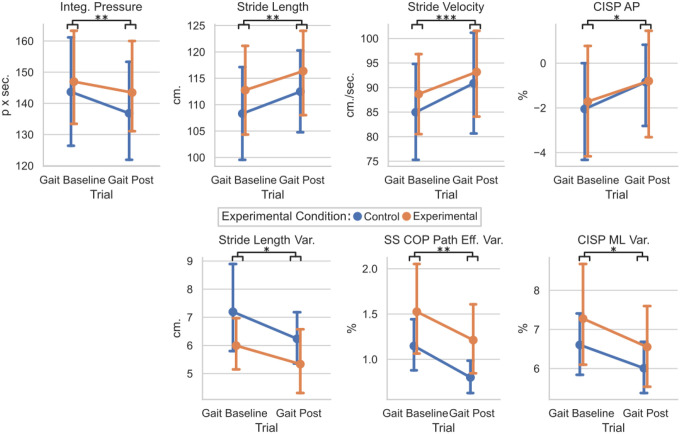
Plot of Gait Baseline vs. Gait Post for the Control and Experimental Groups for Gait Characteristics. Statistically significant values were defined as * : p <0.05, ** : p < 0.01, *** : p < 0.001.

To determine if the entire training altered the unperturbed walking between the EG and CG groups, the means and standard deviations of the Gait Baseline and Gait Posts were compared for each group (Fig. 4). Significant main effect of trials were found for the stride length (F(1,36) = 12.6, p = 0.0011), transformed stride velocity (F(1,36) = 15.0, p < 0.001), transformed integrated pressure (F(1,36) = 11.4, p = 0.002), and CISP AP % (F(1,36) = 5.8, p = 0.021). No significant interaction effects were found for the unperturbed trials and groups. Therefore, all participants took longer, faster strides with a more anterior CISP AP % and a lower integrated pressure during the Gait Post trial, regardless of group placement. For the variability, each participant’s standard deviations were evaluated. Significant main effect of trials were found for the transformed stride length variability (F(1,36) = 4.1, p = 0.0498), transformed CISP ML% variability (F(1,36) = 4.2, p = 0.047), and the SS COP Path efficiency variability (F(1,36) = 11.6, p = 0.0016) with no interaction effect. Therefore, all participants had less variable stride length, SS COP efficiency, and CISP ML % during the Gait Post trial regardless of group placement.

#### 2. Test Trials (Test Pre vs. Test Post)

**Figure 5:**
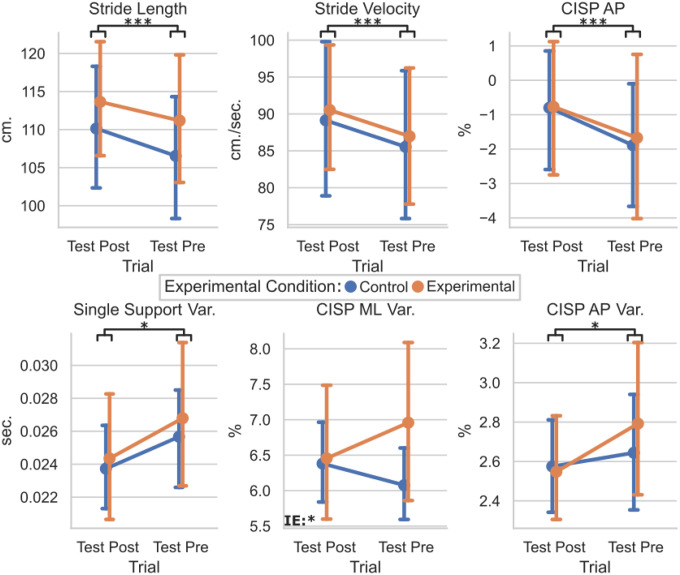
Plot of Test Pre vs. Test Post for the Control and Experimental Groups for Gait Characteristics. Statistically significant values were defined as * : p <0.05, ** : p < 0.01, *** : p < 0.001. Interaction effects, denoted by an IE label in the graph’s lower left corner, indicate statistically significant effects of the session (Pre, Post)×group (EG, CG) interaction term.

To determine if the perturbation training altered the response to the testing perturbations between the EG and CG groups, the means and standard deviations of the Test Pre and Test Posts were compared for each group (Fig. 5). Significant main effect of trials were found for the stride length (F(1,36) = 18.6, p < 0.001) and stride velocity (F(1,36) = 13.0, p < 0.001), and CISP AP % (F(1,36) = 25.6, p < 0.001). No significant interaction effects were found for Test trials and Perturbation groups. Therefore, all participants took longer, faster strides with a more anterior CISP AP % during the Test Post trial regardless of group placement.

For the variability, each participant’s standard deviations were evaluated. Significant main effect of trials were found for the time in single support variability (F(1,36) = 7.2, p = 0.011) and transformed CISP AP % variability (F(1,36) = 4.2, p = 0.49) with no interaction effect. Significant interaction effect was seen in the transformed CISP ML % variability (F(1,36) = 4.3, p = 0.045) with no significant main effect of trial. Therefore, all participants had less variable single support times and CISP AP % during the Test Post trial regardless of group placement. However, the group placement did significantly affect how the CISP ML % variability changed between the Test Pre and Test Post trials.

#### 3. Training Trials

**Figure 6:**
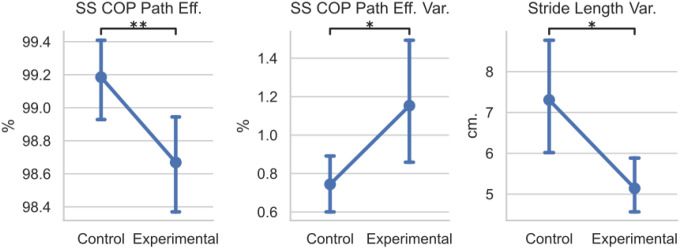
Plot of Training data for the Control and Experimental Groups for Gait Characteristics. Statistically significant values were defined as * : p <0.05, ** : p < 0.01, *** : p < 0.001.

The differences in each participant’s mean and standard deviation are investigated by comparing the EG’s training to the CG’s and shown in Fig. 6. When comparing the two groups means, the SS COP Efficiency was significantly lower in the EG compared to the CG (EG: 98.7+−0.7%, CG: 99.2+−0.6%, U = 278, p = 0.0046). The SS COP Efficiency variability was also significantly higher in the EG (EG: 1.15+−0.7%, CG: 0.74+−0.3%, t = −2.26, p = 0.030). However, the Stride Length variability was significantly higher in the CG than the EG (EG: 5.14+−1.5cm, CG: 7.31+−3.2cm, U = 265, p = 0.014). Therefore, individuals that experienced the perturbation training had less efficient SS COP trajectories with higher variability, and also walked with less variable stride lengths.

## Discussion

Our study investigated the acute effects of PBT with a single session of mTPAD in healthy, neurotypical adults 50 and older. Our study demonstrated several novel findings: a single session of PBT delivered by the mTPAD led to (1) significantly increased cognitive performance in the EG, in addition, we observed (2) significantly increased functional performance by the EG. This is the first randomized, large group (n = 40) clinical study exploring overground perturbation-based CDRGT. As such, future robotic gait trainers that support patients in gaining or regaining their balance may be developed with the help of the research from our study.

The study provides further evidence of acute cognitive improvements after a single session of PBT and aligns with some of the results in Martelli et al. (2021) [10]. Specifically, our results revealed that the EG completed the SMDT-C in significantly less time than the CG (Fig. 3B). We agree with the explanation Martelli et al. (2021) [10] provided that describes the reasons for the observation. In response to perturbations delivered while walking, the base of support changes rapidly, requiring spatial navigation, coordination, and physical affordances [49]. To maintain balance, sensorimotor and cognitive processing are necessary for specific domains depending on the task’s type and complexity. We also hypothesize that exposure to perturbations is related to increased activation of cognitive control processes, particularly domains dedicated to the integration of motion and processing speed [2], [50], [51], [52], [53]. Thus, this mode of cognitive activation in the EG may have continued even without stimulation, allowing for increased speed in the SDMT. Extending from Martelli et al. (2021) [10], we also observed that the EG solved the TMT-B faster than the CG after training, while no significant difference was observed in Martelli et al. (2021) [10]. One possible explanation for this difference is that our study benefited from a larger sample size (n = 40 vs. n = 28) and thus increased statistical power to detect acute, subtle changes. In addition, the inclusion of the MOCA provides a means to screen for underlying cognitive impairments that were not performed priorly. All participants scored within the normal range for the MOCA, with no significant difference between the two groups (Table 2). Furthermore, new evidence points to the validity of the TMT or variants to assess motor-cognitive performance in individuals [54], [55]. Also included in our study was the collection of vitals. Obtained vitals provides evidence that perturbations experienced by the EG did not significantly increase participants’ symptomatic response and arousal than the CG, and thus likely would not be a major contributing factor to observed differences in the EG and CG [56]. We noted that vitals for both the EG and CG, while significantly elevated upon the mTPAD, were not significantly different between the EG and CG (Fig. 3A).

Our results demonstrated that the EG was better adapted to functional tasks after the deliverance of PBT with the mTPAD and that participants of the EG showcased increased confidence in mobility (Fig. 3C). This was evident by participants in the EG standing longer on the 4-Stage Balance Test (one-foot stand) and achieving a higher score on the BBS, which assesses balance through a series of functional tasks. In addition, the EG displayed decreased self-reported concerns about falling after their session as measured by the FES-I.

While there is significant visible variation within the Day 1 and Day 2 clinical test results, the differences in gait characteristics had fewer interaction effects between the group placement and conditions. When walking without force, all study participants took longer, faster strides in the Gait Post session compared to the Gait Baseline. All participant’s CISP AP values were significantly more anterior. This suggests that after undergoing the entire protocol, all participants’ single stance COP trajectory progressed further along the foot, bringing the CISP AP forward. This, along with the significant decrease in integrated pressure, could be tied to increased stride length and velocity, as the lengthening of the stride could have forced the COP further forward along the foot while in a single stance. The variabilities of the stride length, CISP ML, and SS COP path efficiency decreased in Gait Post, indicating that these measures were more repeatable at the end of the protocol. No interaction effects were found for any variable, illustrating that the Experimental condition did not alter any gait characteristics during unloaded walking.

Similar effects were found when comparing the Test Pre and Test Post conditions. All participants took longer, faster strides with a less posterior CISP AP during the second round of lateral perturbations. These changes mirror the changes made from Gait Baseline to Gait Post, which may indicate that participants were more comfortable walking in the mTPAD as the protocol went on. This increase in stride velocity could be a benefit to the individuals as a result of the entire protocol, as slower dual-task walking in older adults has been associated with higher risks of falls [57], [58]. In the second half of the protocol, all participants walked faster, elongating their strides. All participants also had less variable single support time and CISP AP values, supporting that their gait became more regular as they walked. However, the CISP ML variability had a significant interaction effect. While the control group’s ML CISP variability increased slightly from Test Pre to Test Post, the experimental group’s ML CISP variability decreased in Test Post. It is possible that the control group, after walking without perturbations for 10 minutes, were more affected by the lateral perturbations. Therefore, the ML CISP was less variable for those who experienced other perturbations during the training session and more for those who did not experience training perturbations. This exciting result highlights the mTPAD perturbations’ ability to train individuals to withstand variability in the ML CISP caused by lateral perturbations.

While the training perturbations caused a decrease in the ML CISP variability during the lateral perturbations, there were also some differences in the training session between the control group and the experimental group. During the training session, the experimental group had significantly less efficient SS COP paths with higher variability and less variable stride lengths than the control group. Having a less efficient COP path with higher variability illustrates that the random perturbations could introduce variability and alter the COP progression of the individuals in the experimental group, which was one goal of this experiment. This significant decrease in efficiency and increase in variability also highlight the efficacy of the mTPAD as an overground perturbation platform. This increase in COP path deviation may be related to the clinical improvements seen by the experimental group, as this is the critical difference between individuals in the EG and CG.

Although the mTPAD is a fully contained, portable, low-cost robotic trainer, it has limitations. The mTPAD’s compact nature limited perturbations to ~ 10% BW. A more rigid frame and powerful motors could increase ground reaction force. Further studies should assess whether acute aftereffects of PBT via the mTPAD produce long-term changes in gait stability and cognitive performance and can be explored by implementing multiple-session training and follow-up. Such a study would benefit from an even larger sample size and a broader range of older adults. Furthermore, although we observed significant changes in the SMDT-C and TMT-B, which assess cognitive performance, and the 4-Stage Balance Test, BBS, and FES-I, which serve as functional measurements, several cognitive or functional measurements were not significantly different (Fig. 3A–C). We observed no significant difference in both the main and interaction effect of the following (1) cognitive measurements (SMDT-60, SMDT-90, and TMT-A) and (2) functional measurements (SPPB, 4-Stage Balance Test: Feet Side-by-Side, 4-Stage Balance Test: Semi-tandem Stand, 4-Stage Balance Test: Tandem Stand). Explanations to these observations are (1) regarding non-significant cognitive measurements, the SMDT-60 and SMDT-90 represent the number of correct responses in 60 seconds and 90 seconds, respectively, while the SMDT-C represents the completion time in seconds. The difference in range for the SMDT-60 and SMDT-90 were 41 and 61, respectively, versus 260 for the SMDT-C. The SMDT-60 and SMDT-90 do not represent the completion stage, this inherently leads to the SMDT-60 and SMDT-90 being less sensitive benchmarks in teasing out subtle differences. A similar consideration should be made when comparing TMT-A and TMT-B. Participants are scored on the completion time and asked to draw a single line to numbered circles for the TMT-A (e.g., 1-2-3-4-5-6). In contrast, participants must alternate between numbers and letters (e.g., 1-A-2-B-3-C) for the TMT-B, and thus more challenging and sensitive. Given the increased complexity, TMT-B requires more time for completion [59] as the TMT-B is viewed as a measurement of higher-level cognitive ability [60]. (2) Regarding non-significant functional measurements, when comparing the SPPB to the BBS, both assessments of functional tasks, the SPPB has a low diagnostic value in detecting acute, incremental changes to predict negative health-related outcomes balance [61]. For the 4-Stage Balance Test, participants perform four progressively challenging positions starting with a two-feet stand to a one-foot stand. We observed a significant difference in the interaction effect between the EG and CG with the one-foot stand, the most challenging and, thus, the likely most sensitive benchmark. Although no study has comparatively validated each stage individually, single leg stance has often been shown sensitive to detect a significant change in a clinical setting [62], [63], [64].

While further investigation is needed to fully elucidate the benefits and limitations of mobile perturbation-based robotic gait training technology, current evidence indicates that this technology represents a valuable tool in rehabilitation. The technology’s versatility makes it adaptable to various clinical settings, including inpatient rehabilitation facilities, outpatient clinics, and home environments. Another benefit is that the technology can be employed cheaply and function in low-resource settings. Moreover, a key advantage is its capacity to provide consistent and precise feedback to patients during their training sessions which can be tailored to the individual’s specific needs. In conclusion, mobile perturbation-based robotic gait training technology shows great potential in improving individuals’ mobility, balance, and cognitive affluence.

## Figures and Tables

**Table 1:** Tabulation of the Experimental Protocol for Day 1 and Day 2

Day 1	Day 2
Consent	mTPAD
**Interview/Socio-demographics**	**Functional Measurements**
**Anthropometries**	BBS
Height	SPPB
Weight	FES-I
Hip Width	4-Stage Balance Test
Hip Depth	**Vitals**
**Functional Measurements**	Blood Pressure
Berg Balance Scale (BBS)	Heart Rate
Short Physical Performance Battery (SPPB)	**Cognitive Measurements**
Falls Efficacy Scale International (FES-I)	SMDT
4-Stage Balance Test	TMT
**Vitals**	
Blood Pressure	
Heart Rate	
**Cognitive Measurements**	
Symbol Digit Modalities Test (SMDT)	
Trail Making Test (TMT) Parts A & B	
Montreal Cognitive Assessment (MoCA)	

**Table 2:** Descriptive Statistics of Participants’ Baseline Characteristics, Socio-Demographics, Anthropometrics, Vitals, Functional Measurements, and Cognitive Performance in the Experimental Group (EG) and Control Group (CG). Continuous variables are reported as mean ± SD, while categorical variables are reported with number and percentage of the total, n (%).

	Experimental Group (n = 20)	Control Group (CG) (n = 20)	p-value
**Baseline Characteristics**			
Age, Years	71 (± 8)	71 (± 8)	0.906
Female	15 (75%)	15 (75%)	1.000
Increased Fall Risk	17 (85%)	18 (90%)	0.632
Subjective Health (1–5)	4.0 (± 0.6)	4.1 (± 0.7)	0.479
**Socio-Demographics**			
Education (≥ High school)	19 (95%)	17 (85%)	0.291
Employed	9 (45%)	9 (45%)	1.000
Housed	20 (100%)	20 (100%)	1.000
Living Alone	9 (45%)	10 (50%)	0.752
**Anthropometrics**			
Height, cm	164 (±12)	165 (± 8)	0.815
Weight, kg	72 (±17)	71 (±17)	0.926
Hip Width, cm	36 (± 4)	37 (± 5)	0.324
Hip Depth, cm	30 (± 4)	27 (± 4)	0.154
**Functional Measurements**			
Berg Balance Scale (BBS, 0–56)	48 (± 3)	48 (± 3)	0.854
Short Physical Performance Battery (SPPB, 0–12)	11.9 (± 0.4)	12.0 (±0.2)	0.304
Falls Efficacy Scale International (FES-I; 16–64)	38 (± 8)	37 (±11)	0.780
4-Stage Balance Test (0–10 seconds)			
*Feet Side-by-Side*	10 (±0)	10 (±0)	1.000
*Semi-tandem Stand*	9.8 (± 0.6)	9.9 (± 0.4)	0.560
**Baseline Characteristics**			
*Tandem Stand*	8.8 (±1.8)	8.5 (±2.0)	0.681
*One Foot Stand*	4.8 (±3.0)	5.1 (±2.6)	0.740
**Vitals**			
Systolic Blood Pressure, mmHg	113 (±17)	111 (±16)	0.703
Diastolic Blood Pressure, mmHg	76 (± 18)	76 (± 8)	0.954
Heart Rate, beats per minute	76 (± 10)	72 (± 13)	0.413
**Cognitive Measurements**			
Montreal Cognitive Assessment (MOCA)	28 (±1)	28 (± 2)	0.224
Symbol Digit Modalities Test (SMDT)			
*Number Completed in 60 seconds*	28 (± 10)	29 (± 8)	0.589
*Number Completed in 90 seconds*	44 (±12)	44 (±11)	0.946
*Time to Complete*	194 (±39)	190 (± 57)	0.842
Trail Making Test (TMT, Part A & B)			
*Part A seconds*	44 (± 26)	42 (± 17)	0.762
*Part B, seconds*	98 (±30)	97 (± 55)	0.971

## Data Availability

The datasets generated during and/or analyzed during the current study are available from the corresponding author on reasonable request.
